# Preclinical and Clinical Sex Differences in Antipsychotic-Induced Metabolic Disturbances: A Narrative Review of Adiposity and Glucose Metabolism

**DOI:** 10.20900/jpbs.20190013

**Published:** 2019-08-29

**Authors:** Laura N. Castellani, Kenya A. Costa-Dookhan, William B. McIntyre, David C. Wright, Stephanie A. Flowers, Margaret K. Hahn, Kristen M. Ward

**Affiliations:** 1 Centre for Addition and Mental Health, Toronto, ON M5T1L8, Canada; 2 Institute of Medical Science, Faculty of Medicine, University of Toronto, Toronto, ON M5S3K1, Canada; 3 Human Health and Nutritional Sciences, University of Guelph, Guelph, ON N1G1Y2, Canada; 4 Department of Pharmacy Practice, University of Illinois, Chicago, IL 60607, USA; 5 Department of Psychiatry, University of Toronto, Toronto, ON M5T1R8, Canada; 6 Banting and Best Diabetes Centre, University of Toronto, ON M5G2C4, Canada; 7 Department of Clinical Pharmacy, University of Michigan College of Pharmacy, Ann Arbor, MI 48109, USA

**Keywords:** visceral adipose tissue, insulin resistance, metabolic side effects, sex differences, antipsychotics

## Abstract

Antipsychotic (AP) medications are associated with an increased risk of developing metabolic side effects including weight gain, type 2 diabetes (T2D), dyslipidemia, and hypertension. In the majority of clinical studies, females on APs are noted to gain more weight, and are more likely to be diagnosed with metabolic syndrome when compared to males. However, the data is less clear when comparing sex disparities associated with other specific AP-induced metabolic risk factors. Accumulating evidence has demonstrated a role for AP-induced adipose tissue accumulation as well as whole body glucose dysregulation in male models that is independent of changes in body weight. The purpose of this narrative review is to explore the susceptibility of males and females to changes in adiposity and glucose metabolism across clinical and preclinical models of AP treatment. It is important that future research examining AP-induced metabolic side effects analyzes outcomes by sex to help clarify risk and identify the mechanisms of adverse event development to improve safe prescribing of medications.

## INTRODUCTION

Antipsychotics (APs), particularly so called “second” (SGA), and now “third” generation agents, are pivotal in the treatment of schizophrenia and bipolar disorder. They are also being used at increasing rates in patients with depression [1], off-label for conditions such as sleep disorders [2], and for behavioral regulation in children and adolescents [3,4].

The rising prevalence of AP use is concerning for the severe metabolic side effects associated with these drugs. It is well established that AP medications contribute to high rates of metabolic co-morbidity in patients with severe mental illness, in addition to the pre-existing intrinsic metabolic risk attributable to psychiatric illness. For example, in patients with first episode schizophrenia, significant weight gain of at least 7% baseline body weight has been reported to occur in up to 86% of patients taking the SGA olanzapine within the first year of treatment [5]. Moreover, the use of APs has been independently associated with the development of Type 2 diabetes (T2D) [6]. When looking at patients with schizophrenia without age restrictions, the rate of T2D is an astounding 3–5 times higher than that of the general population [7]. Rates of glucose intolerance have been reported to exceed ~55% in young patients with schizophrenia spectrum disorders on APs [8]. Moreover, the incidence of T2D is higher in AP-treated youth compared to untreated individuals with a psychiatric diagnosis as noted by a cumulative odds of T2D diagnosis of 2.09 (95% CI 1.50–52.90; *P* < 0.0001), suggesting that the metabolic risk accrued because of these agents occurs early on in illness [9]. Similarly, in a large cohort of patients in the Danish Psychiatric Central Research Register, patients with schizophrenia who were treated with an AP were at significantly greater risk of developing diabetes when compared to AP-naïve patients with schizophrenia, even after adjusting for confounders (hazard ratio 3.06; 95% CI 1.32–7.05) [10]. Healthy individuals treated in the short term with a standard dose of APs also show similar susceptibility to disturbances in glucose metabolism [11,12].

The goal of this review is to summarize preclinical and clinical literature that has compared sex differences in select adiposity measures and glucose metabolism. We will describe rates of total weight gain, measures of adiposity, and glucose metabolism by sex, while highlighting proposed mechanisms for all disparities.

## METHODS

To identify relevant literature, PubMed was searched using metabolic adverse drug event terms paired with generic drug names or medication classes. Example searches included *“drug name* AND sex OR gender AND weight gain”, “APs AND adiposity”, “APs AND metabolic syndrome” and “insulin resistance AND APs”. Given the abundance of work exploring AP-induced glucose dysregulation, weight gain, and changes in adiposity we chose to focus on the influence of sex on these outcomes. Although lipid metabolism was not a focus of this review a brief search was conducted using the search terms “APs AND lipid metabolism” and “sex differences AND lipid metabolism”. Research exploring AP-induced dyslipidemia with respect to sex differences was limited. Moreover, rodents do not appear to be a good model for AP-induced dyslipidemia, with most available studies failing to find effects of subchronic olanzapine treatment on the lipid profile of rodents [13–16], at odds with what is observed clinically. As such, the topic of dyslipidemia was not included as a focus of the current review.

The use of the search term “gender” was included to capture literature that may have inappropriately classified sex differences as gender differences. Citation lists from critical studies were also reviewed for additional references. Resulting literature is presented below as preclinical (animal studies) or clinical (observational or prospective human subjects studies).

## RESULTS

### Sex Differences and Total Body Weight Gain Associated with AP Exposure

#### Clinical Data

A large body of clinical research has associated female sex with an increased risk of weight gain due to APs [17–25] (Table 1A). Some of these studies have enrolled patients on either clozapine or olanzapine, the two APs known to be associated with the highest rates of weight gain. More recently, Lau *et al.* retrospectively compared weight gain among 110 patients (average age of 34.5) with schizophrenia taking clozapine [17]. They identified female sex as a risk factor for significant weight gain when measured at 3 to 12 months into therapy. There was a +5.5% body weight increase in females *vs* a +1.3% body weight increase in males, *P* = 0.01) [17]. These results are in agreement with a study from 2004 that compared weight gain among patients randomized to open-label clozapine *vs* continuation with a first generation AP (FGA) [18]. Patients on clozapine gained more weight than patients maintained on a FGA over a 2-year period, and the weight gain was significantly higher in women. Data retrospectively analyzed from 3826 patients involved in olanzapine clinical trials also identified female sex among other risk factors (e.g., younger age, and lower baseline body mass index [BMI]) for increased weight gain associated with olanzapine use [24].

In contrast, several studies have found no association between female sex and increased risk of weight gain, or even higher rates of weight gain in men following AP exposure (Table 1A). In a recent study among 119 patients between the ages of 8–19 years of age with schizophrenia or schizoaffective disorder, sex was not found to be a predictor or moderator of weight change over 8 weeks of receiving an SGA [26]. In 2017 Susilova *et al.* observed that men were more likely to gain weight than women when comparing 462 patients (average age of 30 in males and 38.3 in females) with schizophrenia or schizoaffective disorder in the Czech Republic [27]. Similar results were identified in a study of 123 Han Chinese patients with schizophrenia (average age of 34) treated for up to 42 days with risperidone [28]. There is significant heterogeneity in trials with respect to several factors including study population, duration of current treatment, specific agent used, and past medication exposure that may explain the conflicting results.

#### Preclinical data (AP-induced changes in body weight)

In contrast to clinical findings, preclinical work in rodent models has shown more consistent sex-specific differences in the weight gain response to AP exposure. Specifically, repeated treatment with olanzapine has been consistently shown to increase body weight in female [33–46], but not male [33,43,47–55] rats compared to vehicle treated animals. Indeed, in female rats changes in body weight are apparent within approximately 1 week of olanzapine treatment [34,40,46], with some changes reported as early as 24 to 48 h after the initial dose, and coinciding with increases in food intake [33].

##### Female models

###### Olanzapine treatment in female rats

Considerable work has been conducted exploring the effects of route and dose of olanzapine administration on adverse metabolic effects. Repeated olanzapine dosing (*i.e*., 7 days to 13 months) has been shown to increase body weight in female rats across numerous modes of dosing and routes of administration (Table 2A). Olanzapine administered via subcutaneous minipumps for 14 and 30 days has been shown to increase body weight in female rats at doses ranging from 4 mg/kg/day to 8 mg/kg/day [41,42]. Many studies have similarly shown a potent effect of orally administered olanzapine, both via oral gavage and mixed in with food, in increasing body weight at doses and durations spanning 1.2 mg/kg/day to 20 mg/kg/day for 10 to 20 days respectively [33,34,43,45,56]. These effects are consistent with reports demonstrating increased body weight in female rats in response to treatment with intramuscular depot administration of olanzapine with exposure periods of 25 to 35 days [37,44,46,57]. Intraperitoneal olanzapine administration has been shown to induce significant body weight gain as well [35,38,39].

Interestingly, a sensitization effect was noted by Albaugh *et al.* 2006, who reported that body weight plateaus after 7 days of oral olanzapine treatment (4 mg/kg) in female rats [33]. This effect was overcome when olanzapine was increased weekly to a final dose of 20 mg/kg for 20 days of treatment.

While olanzapine has been shown to increase body weight across a wide range of dosing, it is worth noting that an acute dose of approximately 1–2 mg/kg body weight, or a chronic infusion of approximately 7.5 mg/kg body weight in rodents is thought to represent clinically relevant dosing, in that approximately 60%−70% of dopamine receptors are occupied at this concentration [58].

###### Olanzapine treatment in female mice

Interestingly, unlike female rats, female mice did not show changes in body weight in response to 10 day olanzapine treatment increasing from 4 mg/day to 8 mg/day (administered orally) [33]. However, female mice treated with olanzapine for 30 days via osmotic mini-pump did respond with increased body weight [59]. Six-week olanzapine treatment mixed into a high fat diet (45% kcal fat) also increased body weight in female mice allowed ad libitum access to food, however this effect was lost when mice were pair-fed to match food intake of vehicle-fed mice on the high fat diet. Species-specific responses have not been carefully considered but could involve differences in metabolic rate between rats and mice, or differences in hepatic drug clearance. Changes in body composition or metabolic substrates/hormones in response to olanzapine were not assessed in response to the 10-day olanzapine treatment.

###### Clozapine treatment in female rats

In contrast to olanzapine, clozapine had no effect on weight gain, or food intake after 10 days of ramped oral administration (4 mg/kg to final dose of 8 mg/kg) in female rats; however weight gain effects were observed when clozapine was co-administered with olanzapine for an additional 14 day period [33]. In agreement with this work, Cooper *et al.* [36] demonstrated that clozapine alone (administered via intraperitoneal injection) led to decreased body weight in female rats after a 20 day treatment period.

##### Haloperidol treatment in female rats

Although adverse metabolic effects associated with AP use are often attributed to SGAs, metabolic effects are also observed in response to FGAs. Indeed, female rats also showed susceptibility to haloperidol-induced weight gain, in response to oral gavage treatment for 21 days at doses of 0.08 mg/kg/day and 0.31 mg/kg/day [43] but not 10 days at 0.04 mg/kg/day [34].

##### Male models

###### Olanzapine treatment in male rats and dogs

In contrast to preclinical female models, chronic olanzapine treatment (ranging from 7 to 28 days) in male rats has been shown to have no effect on body weight gain [33,48,49,52,53], with some studies reporting decreases in body weight over the treatment period [43,50,51] (Table 2B). This is mirrored by a similar response in male dogs, which did not show changes in body weight or feeding in response to 21-day oral olanzapine treatment [47]. The lack of effect of AP exposure on body weight gain is apparent across treatments, including intramuscular [51] and intraperitoneal [50] injection, oral administration (gavage and mixed in with food) [33,43,48,53] and miniosmotic pumps [49]. Notably, olanzapine-induced increases in body weight are apparent when olanzapine is paired with a “medium-fat diet” (containing 14% proteins, 31% lipids, and 54% carbohydrates) rather than the standard chow diet. In these rats, 3 and 6 week olanzapine treatment resulted in increased body weight and energy intake [52]. This aligns with work by Townsend et al showing high fat diet to independently potentiate acute olanzapine-induced metabolic complications in male mice [61].

###### Clozapine and haloperidol treatment in male rats

The lack of effect of olanzapine on weight gain in male rat models was consistent with a lack of effect of other APs. While 28 day treatment with haloperidol (0.25 mg/kg/day) or clozapine (10 mg/kg/day) both decreased body weight [54], 3 week haloperidol treatment (1 mg/kg/day) mixed in with food had no effect on body weight [53]. Similarly, 21-day haloperidol treatment failed to alter body weight in male rats at doses that initiated weight gain in female rats [43]. Unlike olanzapine, which elicited a weight gain response when paired with a medium fat diet in male rats, haloperidol treatment did not alter body weight or energy intake compared to control medium-fat fed animals [52].

### Sex Differences in Other Adiposity Measures Associated with AP Exposure

#### Clinical data

As a growing body of research has established in human subject studies, adipose tissue distribution is critically important to diabetes and cardiovascular disease risk [62–64]. Visceral adipose tissue (i.e., fat deposited around the visceral organs) is particularly problematic as it is more metabolically active than subcutaneous adipose tissue and allows entry of free fatty acids directly into the liver via the hepatic portal vein, which can lead to insulin resistance [64].

Studies in humans suggest that males, despite typically gaining less weight when taking APs than females, have greater increases in visceral adipose tissue when using APs (Table 1B). For example, Konarzewska et al. measured visceral obesity in 52 normal weight (BMI < 25 km/m^2^) patients with chronic schizophrenia (average age of 46 [males] and 41 years old [females]) and compared body composition to BMI-matched controls in a cross-sectional study [29]. Inclusion criteria required that participants be on the same AP for the three months preceding the study visit. In this study, the most commonly used APs were olanzapine, then risperidone, followed by haloperidol and clozapine. The team used bioelectrical impedance analysis to calculate body composition. They found that a schizophrenia diagnosis was significantly linked to higher amount of visceral adipose tissue and lower fat-free mass in both sexes. Furthermore, men with schizophrenia averaged at least 5 times, and women with schizophrenia at least 2 times, as much visceral adipose tissue as age-matched controls.

A similar cross-sectional study in Japan compared body composition among patients with schizophrenia to healthy controls [30]. The study team enrolled 204 subjects, with an average age of 39 (males) and 43 (females), and used bioelectrical impedance to assess body composition. Significant baseline differences between the control and schizophrenia groups, by sex, included shorter stature in the schizophrenia groups, greater body weight in the females with schizophrenia, and higher BMI in the male and female schizophrenia groups. The investigators identified higher body fat, higher percentage body fat, lower fat-free mass and muscle mass, and lower body water among males with schizophrenia when compared to controls. In contrast, females with schizophrenia had a significantly lower percent body fat, higher fat-free mass, higher muscle mass, and higher body water than controls. The investigators did not describe the extent and type of AP use among the patients with schizophrenia, but did cite the inclusion of patients taking either first or later generation APs as a potential study limitation.

Zhang *et al.* also compared body composition changes using MRI in 46 patients with schizophrenia who were exposed to their initial AP for 10 weeks with age and sex matched healthy controls [31]. In this study, the average age of participants was 27 years old. Males with schizophrenia had significantly higher visceral and subcutaneous fat distribution after 10 weeks of treatment with either risperidone, chlorpromazine, or quetiapine, when compared to baseline. In females with schizophrenia, there was a significant, but less profound increase in absolute visceral fat distribution than that observed in the males when compared to baseline (i.e., an average increase of 7.8 cm^2^ in women compared to an average of 25.2 cm^2^ gained in men). For both males and females, there were no significant differences between the patients with schizophrenia and the controls.

However, most literature to date comparing body composition between patients with schizophrenia to healthy controls has included all males [32], or did not differentiate results by sex [65–67]. In this regard preclinical data is helpful to elucidate a relationship between sex and body composition changes due to APs.

#### Preclinical data (AP-induced changes in adiposity)

In contrast to the discrepancies previously discussed between preclinical and clinical data with respect to sex differences in total body weight gain associated with APs, preclinical data have largely paralleled clinical data in supporting an increased risk of visceral adiposity in males (Table 2).

##### Female models of AP-induced adiposity

In line with AP-induced weight gain, female rats exposed to chronic olanzapine treatment displayed increased accumulation of visceral adipose tissue depots compared to vehicle treated control animals [40,42,45] (Table 2A). In a 21 day olanzapine treatment (i.p. injections, 4 mg/kg/day) study, medication use was associated with increased intra-abdominal fat deposition [38]. This was confirmed by Cooper *et al.* who similarly demonstrated that 20 day olanzapine treatment increased perirenal fat pad mass alongside increases in body weight and food intake [35]. Similarly, female mice exposed to olanzapine for 30 days displayed increased peri-uterine white adipose tissue mass in parallel with increased body weight [59].

Remarkably, although 20-day clozapine treatment decreased body weight in female rats, this treatment simultaneously increased visceral adiposity, independent of changes in food intake or loss of lean mass. In this study clozapine treatment was also associated with an increase in serum adiponectin, which the authors propose could increase adipose tissue insulin sensitivity and subsequently increase lipogenesis [36]. This work highlights the metabolic susceptibility that occurs even in the presence of decreased body weight or stable food intake.

##### Male models of AP-induced adiposity

The potential for metabolic complications to occur independent of changes in body weight is especially apparent when considering the effects of chronic AP use in preclinical male models of adiposity (Table 2B).

Indeed, despite a lack of effect on total body weight, 3 week haloperidol and olanzapine treatments (mixed in with food) increased subcutaneous and retroperitoneal white adipose tissue in male rats [53]. In line with this study, reports of olanzapine-induced decreases in body weight over a 20 day treatment period were matched by increases in visceral adiposity and reductions in lean body mass [50], accounting, in part, for reductions in body mass. Reports from Ferno *et al.* echo these findings, showing that despite a transient increase in food intake in the first 7 days of olanzapine treatment, total body weight was reduced in the total 17-day treatment period alongside increased mesenteric white adipose tissue and increased liver weights [51]. Male dogs show similar increases in adiposity in response to 21-day oral olanzapine administration despite body weights remaining stable during the treatment period [47].

Interestingly, 28-day haloperidol and clozapine treatment reduced body weight in male rats, however these drugs were not found to effect adiposity. By comparison, 28-day quetiapine treatment did not alter body weight compared to vehicle rats, but increased adiposity by approximately 40% (measured via dual-energy X-ray absorptionmetry [DEXA] scanning) [54]. Extended quetiapine treatment (42 day) was similarly found to increase adiposity, which, in contrast to olanzapine treatment, was not worsened when co-administered with a high fat diet [55].

Ultimately, this literature emphasizes a role for body weight-independent metabolic risk in both male and female models following chronic AP exposure that appears to be conserved across dose, agent, route and species. Given the intimate association between adiposity and other comorbid conditions, such as glucose dysregulation and insulin resistance, it is interesting to consider how these complications might also manifest with sex specific considerations.

### Sex Differences in Glucose Metabolism

In addition to AP-induced changes in adiposity and body weight, APs have been shown to influence glucose dysregulation in individuals with schizophrenia, ultimately promoting the development of T2D in youth [9] and adults [10]. As reviewed by Bergman and Ader, the development of T2D in patients with schizophrenia is independently influenced by many environmental, lifestyle, and genetic factors, making it difficult to delineate the extent of the effects of APs on glycemic risk in this population [6]. Few studies in patients with schizophrenia have examined differences between males and females with schizophrenia with respect to measures of glucose metabolism. In a cross-sectional review of 287 patients with schizophrenia on APs (average age of 47), males had higher fasting glucose than females after those taking olanzapine and clozapine were excluded [68].

Studies examining short-term AP use (independent of chronic adipose tissue accumulation) in healthy individuals have offered insight into the effects of APs on glucose disturbances, independently of factors related to the illness of schizophrenia and adiposity. While these effects have been considered in mixed male and female participants, acute dosing studies examining changes in glucose metabolism have yet to directly analyze sex-specific effects.

#### Clinical data

##### Acute dosing in healthy male and female participants

There is a mix of data on the effects of APs on glucose metabolism in healthy human participants (Table 3). A single oral dose of olanzapine (10 mg/day) was shown to increase fasting blood glucose and decrease glucose effectiveness in 15 male and female (80% male) drug naïve participants, though insulin levels in response to treatment were not reported. There was no significant effect of sex or ethnicity with respect to the outcomes of interest, except in the case of prolactin where the difference in prolactin levels between olanzapine and placebo was significantly higher in women (*t*_12_ = 4.6, *P* = 0.0006) [11]. In line with this work, three-day olanzapine treatment combining males and females (8 males, 7 females) showed worsened glucose tolerance in response to an oral glucose challenge, with no change in serum insulin [12]. In contrast, acute olanzapine treatment has also been shown to have no effect on fasting glucose levels in combination with increases in serum insulin post-treatment [69,70]. A cohort of 13 male and 4 female participants treated with olanzapine (oral, 10 mg/day) for 15 days had no change in fasting blood glucose, but showed increases in serum insulin, c-peptide levels and body weight [70]. Olanzapine treatment did result in an increased insulin response during a hyperglycemic clamp and also was associated with a decreased insulin sensitivity index. However, when BMI was controlled, these olanzapine-induced changes were no longer observed [70]. On the other hand, seven day olanzapine treated was also associated with the development of insulin resistance, despite no change in fasting glucose levels post-treatment [69]. Sowell *et al.* further indicated no effect of 21-day olanzapine or risperidone treatment on insulin sensitivity, insulin secretion or glucose tolerance as assessed by a mixed-meal tolerance test in a study of 64 participants (77.3% males) [71]. Although this work suggests a role for insulin secretion in the initial glycemic response to olanzapine, it fails to delineate sex specific contributions to the metabolic response. Future work directly analyzing the effect of sex in mixed male/female study populations is required to fully understand this relationship.

##### Acute dosing in healthy male participants

Work completed in drug-naïve healthy male participants has shown decreased whole-body insulin sensitivity in response to 8-day olanzapine treatment (10 mg, oral) that was associated with reductions in glucose uptake and/or increases in prolactin but not cortisol levels. Despite no change in adiposity, 10 day olanzapine treatment was associated with an increased homeostatic model assessment of insulin resistance (HOMA-IR) and also with decreased insulin sensitivity [74]. In contrast, Vidarsdottir et al, reported that 8 day olanzapine treatment had no effect on fasting glucose or insulin levels while it increased glucagon concentrations and impaired whole body insulin sensitivity [75]. Unlike 10 day olanzapine treatment which has been shown to decrease whole body insulin sensitivity [73], a single dose of olanzapine (10 mg) did not affect insulin sensitivity or secretion in healthy male participants [72].

### Preclinical Data (AP Treatment and Glucose Dysregulation)

The association between adipose tissue accumulation and the development of insulin resistance has been well established in preclinical and clinical models. It is not surprising then that AP exposure (known to increase adiposity) is linked with impaired insulin sensitivity and glucose intolerance [6]. The effects of APs on glucose dysregulation have been studied using both repeated/chronic and single/acute dosing schedules. Chronic administration of APs such as olanzapine allow insight into the longer-term glucose dysregulation that develops alongside changes in adiposity, and captures changes in whole body energy metabolism over time. In contrast, acute studies afford the opportunity to explore the direct effects of APs on glucose homeostasis, independent of changes in confounding variables such as fat mass or loss of lean mass. Combining these approaches affords the best opportunity to identify mechanisms underlying AP-induced glucose dysregulation and development of T2D.

#### Chronic AP exposure and glucose dysregulation

##### Chronic AP-treatment in male models

The effects of AP exposure are particularly apparent in preclinical models where changes in body composition occur consistently in response to AP administration across dose, duration and route of administration. In line with increased adiposity, chronic AP treatment has been shown to impair glucose tolerance and whole-body insulin sensitivity in male rodents and dogs (Table 2B).

Indeed, despite observing a consistent lack of changes in body weight, repeated AP-treatment has been shown to impair glucose tolerance and whole insulin sensitivity in male dogs, mice and rats. Ader *et al.* demonstrated that 21 day olanzapine treatment in male dogs elevated fasting blood glucose and insulin, decreased hepatic insulin sensitivity and reduced beta-cell glucose stimulated insulin secretion, despite stable food intake and body weight [47]. Similarly, 20-day ramped olanzapine dosing in male rats resulted in impaired insulin and glucose tolerance as well as increased fasting glucose and insulin levels in the presence of stable body weights [48]. Similar to olanzapine treatment, 42-day clozapine or quetiapine treatment in male rats has been shown to reduce glucose tolerance alongside increases in adiposity, which also potentiated glucose intolerance induced via high fat feeding [55]. Clozapine-induced insulin resistance can be observed as early as 5 days after subcutaneous treatment, although changes in body weight were not reported in these animals [60].

##### Chronic AP-treatment in female models

Remarkably, oral olanzapine administration via ramped dosing for 20 days did not affect fasting blood glucose levels or glucose tolerance (as measured by an oral glucose tolerance test) in female rats, although glucose bolus dosing caused increased serum insulin levels which are indicative of the early stages of insulin resistance [33] (Table 2A). In a separate study, fasting blood glucose levels were also unchanged in response to 20 day olanzapine treatment via intraperitoneal injection, however; HOMA-IR was elevated in these animals, suggesting the development of insulin resistance [35]. Similarly, 13 day olanzapine administration by oral gavage was not associated with changes in blood glucose or insulin levels despite increased body weight and adiposity [45]. In line with this work, blood glucose and insulin levels after 20 days of clozapine treatment were not significantly different from vehicle treated female rats, despite increased adipose tissue content [36]. In a significantly longer study, chronic olanzapine treatment via intramuscular injection for an exposure period of 13 months was associated with the development of marked glucose and insulin intolerance [37]. Female mice (who displayed increased body weight and adiposity) treated for 30 days with olanzapine via osmotic mini pumps also showed hyperglycemia and hyperinsulinemia, alongside increased HOMA-IR [59] (Table 2A).

In contrast to male rats and dogs, in whom chronic AP-induced glucose dysregulation is apparent, female rats appear less consistently susceptible to glucose intolerance and hyperglycemia despite increased adiposity and body weight at the onset of AP-treatment. Many have reported that APs impair glucose homeostasis directly, independent of changes in body composition [6]. Given the potent effect of chronic AP dosing on increased adiposity, it is difficult to define the role of APs in the dysregulation of glucose metabolism independent of the diabetogenic effects of obesity. Examining the effects of acute AP treatment on glucose dysregulation can provide better insight into these changes.

##### Acute AP exposure and glucose dysregulation

To dissect “direct” effects of APs on glucose homeostasis, acute dosing paradigms, which avoid changes in adiposity, can be helpful. Interestingly, despite increased risk in female rats to AP-induced body weight gain and food intake, male rats show susceptibility to glucose dysregulation which could be, in part, mediated by increased adiposity or loss of lean mass [50] (Table 4). However, as will be reviewed, acute AP treatment suggests susceptibility of male rats and mice to direct disturbances in glucose homeostasis independent of changes in body composition. On the other hand, female rodents appear less susceptible to acute-AP mediated disturbances in glucose metabolism, although less work has been conducted examining this phenomenon in female models.

##### Acute AP-treatment on changes in blood glucose in male models

A single treatment of olanzapine has been shown to rapidly and significantly increase blood glucose in male mice [61,82–84,89,91] and rats [48,81,85,87,88,92], with peak blood glucose occurring approximately 1–2 h post treatment (Table 4B). Indeed, both a single dose of olanzapine (administered via subcutaneous injection [85], intravenous infusion [79,94], intragastric infusion [87], oral gavage [85]) as well as a twice daily dose via oral gavage (one dose in the evening, one dose one hour preceding measures) [48,81] increased blood glucose in male rats. Similarly, a single injection of olanzapine (intraperitoneal injection [82–84,89] or intracerebral ventricular infusion [79,89,91]) in male mice significantly increased blood glucose levels compared to vehicle treated mice. Interestingly, olanzapine-induced hyperglycemia is more pronounced in the fed compared to the fasted state. An intraperitoneal injection of olanzapine in fed animals resulted in a greater increase in blood glucose the hours post-injection compared to fasted male mice [90]. Similarly, centrally-administered olanzapine only increased blood glucose in fed, but not fasted animals, suggesting a protective role for metabolic factors altered by fasting/feeding in olanzapine-mediated glucose dysregulation [91].

##### Serum insulin levels in male models of acute AP-treatment

Notably, despite rapid increases in blood glucose, serum insulin levels are not consistently altered in either rats or mice treated with olanzapine [48,84,89,87,79] (Table 4B). This raises the possibility that, in male rats, appropriate insulin responses to elevations in glucose are inhibited by APs. This is supported by findings demonstrating olanzapine associated reductions in insulin responses (*i.e*., decreased serum insulin and c-peptide levels) following a bolus of glucose, or during hyperglycemic clamps [85,88,86]. Similarly, serum insulin did not change in response to an oral glucose tolerance test whereas olanzapine treatment worsened glucose tolerance (*i.e*., blood glucose remained significantly elevated over time in response to oral glucose bolus) [81]. Moreover, during a pancreatic-euglycemic clamp during which insulin levels are exogenously infused to basal pre-prandial fed conditions, olanzapine had no effect on insulin sensitivity [93]. This suggests that when pancreatic insulin secretion is artificially maintained the effects of olanzapine on glucose metabolism are minimized. As such the inability of male mice and rats to preserve insulin responses in the face of elevated blood glucose levels may leave them susceptible to AP-induced hyperglycemia.

##### Insulin sensitivity in male models in response to acute AP-treatment

Alongside impaired insulin secretion and elevated blood glucose levels, acute olanzapine treatment has been shown to impair whole body insulin sensitivity. As measured by a hyperinsulinemic-euglycemic (HIEC) clamp, the gold standard technique to assess whole body insulin sensitivity, acute exposure to a subcutaneous bolus of olanzapine significantly decreased insulin sensitivity in male rats [60,81,85,86] (Table 4B). This aligns with work demonstrating worsened insulin tolerance following a bolus of olanzapine in male mice [82,83] and rats [81]. Interestingly, some [79], but not all [88] have shown that central administration of olanzapine also worsens insulin sensitivity in male rats during a HIEC.

Acute clozapine treatment has been similarly shown to impair whole body insulin sensitivity as measured by a HIEC. Treatment with a subcutaneous bolus of clozapine resulted in increased hepatic glucose production, as was noted with olanzapine treatment, in male rats [60,86].

Although insulin resistance is consistently reported in response to acute AP-dosing, it is unlikely that it exists as an initiating factor in glycemic disturbances. Previous reports have instead highlighted an integral role for catecholamines and glucagon in the activation of hepatic glucose production by olanzapine [94,95]. Indeed, olanzapine-induced increases in blood glucose are ablated in glucagon receptor knockout mice, despite the onset of olanzapine-induced insulin resistance (as measured by an insulin tolerance test). In this study, olanzapine also induced an exaggerated rise in blood glucose in response to a bolus of pyruvate in wildtype but not glucagon receptor knockout mice, suggesting increased hepatic gluconeogenesis by olanzapine only in the presence of active glucagon receptors [95]. This aligns with work from Nagata *et al.* who demonstrated that a 30 min pretreatment with beta-adrenergic receptor antagonist propranolol can mitigate olanzapine (i.v. treatment, 10 mg/kg)-induced increases in blood glucose [94]. This highlights the ability to recover glucose homeostasis in the absence of improved insulin sensitivity.

##### Blood glucose and serum insulin levels in female models of acute-AP treatment

In contrast to male models, increases in fasting blood glucose are not as consistently observed in female rats following acute olanzapine treatment [33,80] (Table 4A). Unlike the case in male rats, twice daily acute oral gavage with olanzapine over a 29 h exposure period significantly decreased fasting blood glucose levels in female rats [33]. Similarly, a single dose of olanzapine administered via subcutaneous injection did not alter blood glucose levels one hour post-treatment in females [77,80]. Interestingly, although olanzapine did not raise blood glucose, serum insulin levels were significantly elevated post-olanzapine treatment [77,80]. In contrast, Jassim *et al.* reported blood glucose levels to be increased approximately 1 mmol/L one hour post treatment in female rats, and this occurred in the absence of changes in serum insulin [78]. This finding aligns with work conducted by Martins *et al.* [79] who showed an increase in blood glucose in fasted female C57/BL6 mice treated with an intraperitoneal bolus of olanzapine (4.5 mg/kg). Changes in serum insulin in these mice were not reported. In agreement with this work a single study has shown olanzapine to significantly increase both blood glucose and serum insulin levels in female rats [76]. While this effect is often seemingly more subtle than olanzapine-induced hyperglycemia observed in studies using male mice (increasing approximately 5 mmol/L compared to vehicle treated mice) [82,84,90], further work is required to directly compare changes in blood glucose in male and female preclinical models. It would appear that females may be protected against SGA-induced hyperglycemia, and this may be driven by increased insulin secretion (or responses).

##### Glucose tolerance in female models of acute AP treatment

In comparison to male models, AP-treated female rodents have shown less consistent responses to a glucose challenge (Table 4A). In a study by Albaugh *et al,* acute oral olanzapine (4 mg/kg) administration (in addition to lowering of fasting glucose levels) was associated with improved glucose tolerance, and increases in serum insulin levels during an oral glucose tolerance test [33]. This effect appeared to be dependent on the dose and route of administration of olanzapine. Higher doses (7.5 mg/kg and 15 mg/kg, and 10 mg/kg) administered via intraperitoneal and subcutaneous injections respectively worsened glucose tolerance in response to an intraperitoneal injection of glucose [76,80,77]. The inconsistencies in these results could highlight specific dose and route of administration effects of olanzapine treatment in female rodents.

##### Insulin resistance in female models of acute AP treatment

In line with dose-dependent effects on glucose dysregulation in female rats, Wu *et al.* utilized a hyperinsulinemic-euglycemic clamp (HIEC) to identify significant increases in insulin resistance in response to 15 mg/kg bolus of olanzapine, but found no effect of olanzapine at a dose of 1.5 mg/kg (both treatments administered via subcutaneous injection) [80] (Table 4A). Similar to male rats, glucose dysregulation appears to involve unchecked glucose production, rather than impaired insulin stimulated glucose uptake. Specifically, Houseknecht *et al.* revealed that epitrochlearis muscle incubated *ex vivo* 1 h post-olanzapine treatment (10 mg/kg, subcutaneous injection) from female rats showed basal and insulin-stimulated glucose uptake to be intact [60] (Table 4A).

In summary, acute AP-treatment in preclinical models reveals males are susceptible to dysregulated blood glucose independent of changes in body composition, an effect less consistently observed in female models. These results suggest sex differences in insulin metabolism.

### Potential Mechanisms Underlying Observed Sex Differences in AP-Induced Adiposity and Glucose Dysregulation

A number of potential mechanisms have been proposed to explain adverse metabolic side effects of APs, but few directly address how these mechanisms may differentially affect males and females. Below, we have briefly highlighted four areas of research that consider the importance of sex differences in proposed mechanisms underlying AP-induced metabolic side effects: food intake, energy expenditure, the influence of sex hormones, aging, drug metabolism and the gut microbiome composition.

#### Food intake

Among the several mechanisms involved in regulation of body weight, changes in food intake are considered an integral factor driving AP-mediated changes in body weight, particularly in preclinical studies. Specifically, increases in body weight were consistently matched by increases in food intake in female rats and mice in response to AP treatment [34,35,38,40,42–46,56,57,59], while this feeding effect was absent in female mice [33] and male rats and dogs who remained body weight stable [33,47–50,53]. In male rats, olanzapine treatment has been shown to increase meal size and to decrease feeding rate [23], while olanzapine and clozapine treatment have demonstrated increased preference for fat intake [96], suggesting a hedonic effect of these APs in the absence of changes in overall food intake. In clinical studies, data does support an increase in appetite, motivation to eat, and food consumption associated with APs [97,98], but there has been less attention to the impact of medications on these factors and how they may differ by sex. Factors driving these changes in regulation of food intake remain to be fully understood but could involve changes in insulin action, hormone signaling [99], and/or nutrient sensing [100].

#### Energy expenditure

In addition to changes in food intake, changes in energy expenditure are critical to understanding the effects of APs on whole body energy balance. Indeed, resting energy expenditure was increased in healthy male volunteers treated with olanzapine, although these men also displayed increased food intake and body weight at the end of the treatment period (13 days) [101]. In contrast, a smaller study examining nine participants (6 men and 3 women) demonstrated that approximately 12 weeks of olanzapine treatment had no effect on resting energy expenditure in conjunction with increased body weight [102]. How sex might effect AP-induced changes in energy expenditure has not been well-investigated, but should be considered more carefully in future work. Male and female participants have been previously reported to display differences in resting energy expenditure, however this sex-effect was lost when body weight was controlled for [103]. Moreover sex hormones (e.g., estrogen and testosterone) are well appreciated to be involved in the regulation of resting energy expenditure [104], suggesting that AP-induced perturbations in energy expenditure may differ between men and women.

Preclinical models of AP treatment have shown similar effects on energy expenditure. Female C57BL6 mice fed a high fat diet containing olanzapine for 3 days showed decreased physical activity yet increased heat production and energy expenditure in response to the AP treatment. Resting energy expenditure (RER) was not affected by olanzapine treatment in these mice. This effect was maintained in male C57BL/6 mice who displayed increased energy expenditure and decreased physical activity [105]. Townsend *et al.* similarly showed that oxygen consumption was increased in high fat diet fed male mice treated acutely with olanzapine [61]. Acute olanzapine treatment in male rats was also shown to increase heat production in the light phase (when animals are expected to be sleeping) but not in the dark (active) phase [106]. Changes in energy expenditure could reflect innate metabolic changes in response to AP treatment, but may also be confounded by preclinical housing conditions. Emerging literature has specifically highlighted the metabolic consequences (i.e., increased resting energy expenditure) of housing animals at standard conditions, slightly below thermoneutrality [107,108]. It is possible that olanzapine-induced weight loss in male rodents housed at standard conditions are confounded by increased resting metabolic rates in response to heat production. How housing below the thermal neutral temperatures may affect female rodents differently from males and the consequences for metabolic perturbations such as AP treatment remains to be examined.

#### Sex hormones

Sex hormones are intriguing as potential moderators of adverse metabolic effects due to APs. Most obviously, the decline of estrogen in menopause is known to be associated with decreased subcutaneous adipose tissue and increased visceral tissue in females [109] that mirrors AP-induced changes in adiposity. Reviews by Lizcano and Mauvais highlight the role of estrogen in regulating adipose tissue distribution, appetite, satiety, energy regulation, and fat metabolism [109,110]. A role for estrogen has also been identified for the maintenance of glucose homeostasis [111].

Preclinical models have demonstrated an effect of estrogen on AP-mediated accumulation of adipose tissue, changes in feeding and glucose homeostasis, in ovariectomized rats treated with olanzapine [35,46,112,113]. For example, the AP sulpiride, like olanzapine, has been observed to cause weight gain in female, but not male rats [114]. Shimizu *et al.* expanded upon this observation to measure changes in weight and food intake in female rats post ovariectomy, and again following estrogen supplementation [114]. They found that post ovariectomy, the hyperphagia and weight gain induced by sulpiride pre-ovariectomy was absent, but that it could be restored after 14 days of estradiol injections. Similarly, Skrede *et al.* [46] demonstrated that the orexigenic effects of olanzapine and drug-induced body weight gain were attenuated in ovariectomized rats compared to sham-operated animals. This effect was reversed following 8-day estradiol replacement. A clinical study that included 20 female patients with schizophrenia, taking either olanzapine or risperidone for 3 months, noted a significant correlation between estradiol and increases in BMI. The authors suggested that this supported previous literature suggesting that APs affect the hypothalamic-pituitary-gonadal axis [115]. Together this work identifies estrogen as a factor promoting chronic olanzapine-induced increases in body weight and highlights a role for reproductive status on AP-induced weight gain and/or vice versa.

Finally, estrogen has been shown to intimately regulate glucose metabolism, in part via stimulation of insulin secretion and suppression of glucagon release [116]. It is possible that the dampened effect of APs on acute glucose dysregulation in female rodents could involve the ability of estrogen to enhance glucose-stimulated insulin release [117]. While male mice seem to lack the appropriate ability to release insulin in response to increased glucose levels [84], it is possible estrogen preserves the insulin response in female rodents, though this has not been investigated. Moreover, given the integral role for glucagon action in olanzapine-mediated hyperglycemia [95], estrogen mediated reductions in glucagon secretion [116] could similarly attenuate glycemic dysregulation post-acute olanzapine. More work is required to directly assess the effects of estrogen on AP-induced metabolic deregulation.

#### Aging

The proposed role for estrogen highlights the effect of age and reproductive status on AP-induced metabolic responses. Post-menopause a decline in the response to AP treatment has been reported in women with schizophrenia [118]. Similarly, premenopausal women show significantly better treatment response to olanzapine and haloperidol treatment than post-menopausal women [119]. Elderly individuals also display increased plasma levels of olanzapine (weight-adjusted) compared to younger olanzapine-treated patients, suggesting unique AP pharmacokinetics across the lifespan [120].

Independent of AP exposure, menopausal status is known to dramatically affect metabolic health, including changes in adiposity. While premenopausal women are prone to accumulate subcutaneous and gluteal adipose tissue, men and post-menopausal women accumulate visceral adipose tissue in the abdominal region [121]. Similarly, menopause has been shown to influence the development of type 2 diabetes [122]; an effect which could be potentiated with AP treatment. Moreover, gradual decline of testosterone observed in aging men is similarly associated with distinct alterations in body composition. Specifically, the loss of testosterone is associated with a loss of lean mass, accumulation of central/visceral adiposity, and decreased mobility [123]. How this could interact with APs to influence metabolic health remains to be investigated.

#### Drug metabolism

Sex specific metabolic responses to AP treatment could also reflect well established differences in pharmacokinetics between men and women [124]. Men are reported to have a higher clearance of olanzapine (approximately 38%) compared to women [125] as well as increased activity of cytochrome P450 (CYP) 1A2 (the hepatic enzyme predominantly responsible for olanzapine breakdown to metabolites in the liver) [126,127], which could in part explain sex-specific responses to AP treatment. Regulation of drug metabolism is known to involve rate and mode of absorption, levels of hepatic phase I enzymes, and mode of elimination, all of which are altered by sex and influenced by sex hormones, age and body composition [125,128]. Exogenous increases in estrogen (via oral contraceptive) have shown to significantly decrease CYP 1A2 levels in treated compared to untreated women highlighting the effect of sex hormones on CYP 1A2 activity and risk for decreased AP clearance [127]. How AP-induced changes in body composition and metabolism may further confound innate sex differences in drug metabolism over the course of treatment is not yet known.

#### Microbiome

Another mechanism that may underlie sex differences in AP-induced metabolic disorders receiving increasing attention is gut microbiome dysbiosis (i.e., a pronounced shift and subsequent imbalance in gut microbiota). Recent *in vitro* data demonstrates that both first and second generation APs (despite their differences), exhibit direct activity against common commensal gut microbes [129]. Gut dysbiosis associated with AP treatment has been shown to lead to increased systemic inflammation [130], altered structure and function of the intestinal mucosal barrier [131], and disrupted energy metabolism [132], all of which consequently lead to the development of obesity and metabolic disturbances [133–135]. Recent preclinical research in germ-free mice, or mice raised in a sterile environment, has also noted that the presence of a microbiome is essential for AP-induced weight gain [136,137].

With respect to clinical microbiome research, Bahr *et al.* investigated the association of risperidone and AP induced weight gain in relation to the gut microbiome [138]. They observed that chronic risperidone treatment was associated with an increase in body mass index and a significantly lower ratio of the bacterial phyla Bacteroidetes: Firmicutes, as compared with AP-naïve psychiatric controls. Additionally, Bahr *et al.* found the gut microbiota of participants treated with risperidone to be enriched for pathways implicated in weight gain, such as short-chain fatty acid production. In a separate study comparing the gut microbiota of individuals treated with APs to psychiatric participants treated with mood stabilizers, Flowers *et al.* found AP treatment to be associated with higher body mass index and decreased bacterial species richness in feces [139]. AP treatment was also associated with medication-specific decreases in *Akkermansia muciniphilia,* a bacterial species routinely associated with anti-inflammatory properties and metabolic protective effects [140]. Finally, a study by Yuan *et al.* found risperidone treatment over the course of 24 weeks to results in a significant increase in the number of fecal *Bifidobacterium* spp. and *Escherichia coli* in study participants. This was associated with obesity [141].

With regards to the role of sex in this interplay between metabolic disturbances and gut microbiome-associated dysbiosis, both Yuan *et al.* [28] and Flowers [139] did not observe different metabolic outcomes between males and females, while Bahr *et al.* only assessed adolescent male participants. However, an additional study conducted by Flowers *et al.* in 2019 that recruited patients with schizophrenia or bipolar disorder did observe decreased species richness in females on SGAs compared to females on mood stabilizers. This relationship between species richness and APs was not observed in males [142]. Due to the limited studies assessing this interrelationship, further investigation is needed to better explain the influence of sex on the interaction between AP-associated microbiome dysbiosis and metabolic response.

## CONCLUSIONS

In summary, we reviewed observed associations between sex, adiposity, and changes in glucose metabolism as associated with AP use in preclinical and clinical studies. Despite some disagreement among clinical data, female sex was more often associated with greater weight gain in patients on AP in clinical research studies, and this finding was consistently replicated in preclinical research with rodents, particularly upon exposure to olanzapine. With respect to changes in fat distribution patterns following AP exposure, there is a small body of clinical research studies suggesting that, despite females gaining more total weight due to APs, males are more likely to accumulate metabolically adverse visceral adipose tissue when treated with APs. In preclinical models, this is supported by AP-related increases in visceral fat mass, despite reports of weight loss or lack of weight gain in male models. Much of our discussion on AP-related disturbances in glucose homeostasis stemmed from preclinical studies, which suggest that males could be more susceptible to glucose dysregulation independent of changes in body composition as compared to females. This however remains to be replicated in humans. Proposed mechanisms underlying the differential metabolic responses by sex to APs include variable changes in food intake patterns, effects on the microbiome, and the influence of sex hormones. The preclinical and clinical research studies considered here identified sexually divergent metabolic responses to chronic and acute AP treatment though both sexes suffer from adverse metabolic complications.

## Figures and Tables

**Table 1.** Clinical study highlights examining weight gain and adiposity changes associated with antipsychotics in patients with serious mental illness.

A. Weight Gain Associated with Antipsychotics			
AUTHORS	PARTICIPANTS	AP TREATMENT	METABOLIC OUTCOME
Lau *et al.* (2016) [17]	• Retrospective Study • Males (*n* = 67) + Females (*n* = 50) • Mean age: 34.5	• Clozapine use for at least 1 year (Compared 3 month to 12 month treatment time points) • Average clozapine dose of ~300 mg (3 months) and ~316 mg (12 months)	• Females gained significantly more body weight during 9 month clozapine treatment (5.5% weight increase in females *vs* 1.2% increase in males)
Covell *et al.* (2004) [18]	• Patients with schizophrenia • Non-responsive to treatment with 2 other APs • Males (*n* = 43) + females (*n* = 60)	• Switched to clozapine and monitored for 2 year treatment • Control patients remained on “usual care”	• Significantly more women (28%) become obese compared to men (3%) treated with clozapine • Significantly more women (29%) gained more than 20% baseline weight compared to men (13%) over 2 year clozapine treatment period
Xiang *et al.* (2011) [19]	• Retrospective survey • Inpatients with schizophrenia • Males (3734) + females (2707) • Mean age: 44.0	• 3 months of AP treatment	• Significantly more female patients recorded complaints of weight gain compared to males
Hakko *et al.* (2006) [21]	• Longitudinal study • Males + females • Age: Birth-31 years • Males (2947) + Females (3026)	• BMI measured at 14 and 31 years of age • Lifestyle and health questionnaire administered at 31 years of age to assess variables that included medication use and diagnoses, but ultimately there was not enough data to include antipsychotic use information in model	• Females with a psychiatric disorder were significantly more likely than men to transition from under or normal weight to overweight or obese (3.6 fold risk females *vs* 2.1 fold risk in males)
Verma *et al.* (2009) [22]	• Prospective and naturalistic study • First-episode psychiatric disorders with no prior AP exposure • Males (*n* = 29) + females (*n* = 27) • Mean age: 29.8	• 6 month AP treatment • Included mono and dual therapy (60.7% received an FGA, 62.5% received an SGA)	• BMI was significantly increased following 6 month treatment • Female sex was a significant predictor of weight gain
Lee *et al.* (2004) [23]	• In-patients diagnosed with schizophrenia • Males (*n* = 24) + females (*n* = 48) • Mean age (olanzapine): 33.9 • Mean age (risperidone): 36.3	• Olanzapine treatment average 103.5 weeks, average dose 12.4 mg/day • Risperidone treatment, average 93.2 weeks, average dose 4.5 mg/day	• Olanzapine and risperidone treatment resulted in weight gain • No effect of gender on weight gain risk
Lipkovich *et al.* (2009) [24]	• Patients diagnosed with schizophrenia, schizophrenia spectrum disorders, bipolar mania, bipolar depression, or borderline personality disorder (*n* = 3826) • Mean age: 38.8	• 30 week olanzapine treatment	• Female sex was significantly associated with probability of severe weight gain
Attux *et al.* (2007) [25]	• First episode psychotic outpatients • Males (*n* = 26) + females (*n* = 18) • Mean age:26.3 • (females significantly older than males; mean age 32.8 *vs* 21.9)	6 month antipsychotic treatment	• Both males and females exhibited increased BMI and weight gain • Only females showed increased waist circumference
Taylor *et al.* (2018) [26]	• Youths with schizophrenia or schizoaffective disorder • Males (*n* = 47) + females (*n* = 22) • Mean age: 14.15	8 weeks of antipsychotic treatment with molindone, risperidone, or olanzapine	Sex did not predict or moderate weight change or percent weight change
Susilova *et al.* (2017) [27]	• Retrospective study • Inpatients with a diagnosis of schizophrenia or schizoaffective disorder • Males (*n* = 466) + females (*n* = 271) • Mean age: 30 (males), 38.3 (females)	• Average length of hospital stay range 29.4 days-41.8 days • Received AP monotherapy and polytherapy	• Greater increase in BMI and weight gain in response to polytherapy in men but not women • No significant difference in weight changes between males and females in response to some atypical APs • Significant increase in BMI in men but not women in response to multi-receptor APs and aripiprazole
Lane *et al.* (2006) [28]	• AP naïve patients with schizophrenia • Male (*n* = 68) + female (*n* = 55) • Mean age: 34.0	• 42 day risperidone monotherapy	• Male patients gained more weight than females (by an average of 0.650 kg)
B. Adiposity Changes Associated with Antipsychotics			
AUTHORS	PARTICIPANTS	AP TREATMENT AND ADIPOSITY MEASUREMENT APPROACH	METABOLIC OUTCOME
Konarzewska *et al.* (2014) [29]	• Normal weight patients diagnosed with schizophrenia and BMI-matched controls • Patients with schizophrenia: males (*n* = 33) + females (*n* = 19) • Healthy controls: males (23) + females (22) • Mean age: 42.13	• At least 3 months of continuous treatment with an AP prior to beginning the study, and total use of at least 1 year, for patients with schizophrenia • BIA	• Males and females with schizophrenia had significantly increased visceral adipose tissue mass compared to healthy controls • Men with schizophrenia had significantly greater visceral adipose tissue compared to females with schizophrenia (~5-fold)
AUTHORS	PARTICIPANTS	AP TREATMENT	METABOLIC OUTCOME
Sugawara *et al.* (2012) [30]	• Outpatients diagnosed with schizophrenia • Males (*n* = 74) + females (*n* = 130) • Mean age: 41.3	• AP use was defined by chlorpromazine equivalents, but type and duration of AP was not defined • BIA	• BMI was increased in males and females with schizophrenia compared to healthy controls • Females with schizophrenia had greater body weight, body fat, fat-free mass, muscle mass, and body water compared to healthy females • Males with schizophrenia body fat, and % body fat were significantly higher (while lean mass, and body water content were higher in health males)
Zhang *et al.* (2004) [31]	• AP-naïve patients with schizophrenia • Male (*n* = 27) + women (*n* = 19) • Mean age: 26.9	• 10 week AP treatment • MRI	• 10 week treatment with risperidone, chlorpromazine, or quetiapine increased visceral and SQ fat in males • Adipose tissue gain was attenuated in females compared to males
Satoh *et al.* (2007) [32]	• Males with schizophrenia • *n* = 80 (+ 64 healthy controls)	• All patients with schizophrenia were on an AP (duration not defined) • BIA	• Significantly increased body fat and lower intracellular fluid in men with schizophrenia • Total body fluid decreased in men with schizophrenia

AP: antipsychotic, BIA: bioelectrical impedance analysis, MRI: magnetic resonance imaging.

**Table 2.** Chronic preclinical studies examining antipsychotic effects on weight, adiposity and glucose metabolism.

A. Female Models			
STUDY	MODEL	AP DOSE	EFFECT OF WEIGHT GAIN, FOOD INTAKE, ADIPOSITY METABOLISM
Albaugh *et al.* (2006) [33]	• Female C57Bl/6J and A/J mice • Female Wistar and Sprague-Dawley rats	• Olanzapine (mice); oral; ramped dosing 4–8 mg/kg; 10 days • Olanzapine (rats); oral; 4 mg/kg-20 mg/kg; 20 days • Clozapine (rats); oral; 4 mg/kg-8 mg/kg; 12 days • Clozapine + olanzapine (rats); oral; 4 mg/kg clz- 8 mg/kg clz + 4 mg/kg ola 27 days	(Olanzapine 10 days mice): • No significant change in body weight (trend for weight loss, and decreased food intake) (Olanzapine 20 days rats): • Increased body weight (plateau at 710 days without ramped dosing) • Increased food intake (starting after 24 h) • Increased fasting insulin levels (not fasting glucose) • No effect on glucose tolerance, increased insulin during OGTT (Clozapine − rats): No effect on body weight, food intake (Clozapine + Olanzapine-rats): Increased weight gain, increased food intake
Arjona *et al.* (2004) [34]	Female Sprague-Dawley rats	• Olanzapine; oral gavage; 1.2 mg/kg/day; 10 days • Haloperidol; oral gavage; 0.04 mg/kg/day; 10 days	• Olanzapine: Significant increase in body weight and food intake • Decreased total motor activity • Haloperidol: No effect on body weight or food intake
Coccurello *et al.* (2009) [59]	Female CD-1 mice	Olanzapine; 30 day mini-osmotic pump infusion; 4 mg/kg, 8 mg/kg	• Weight gain, hyperphagia, hyperglycemia, hyperinsulinemia, • Increased pancreatic insulin, increased insulin resistance, *i.e.,* increased HOMA-IR • Increased periuterine adipose tissue • Decreased RER, decreased metabolic rate
Cooper *et al.* (2005) [35]	Female Han Wistar rats	Olanzapine; I.P.; 1,2,4 mg/kg/day; Twice daily, 20 days	• Increased body weight at 1 and 2 mg/kg (not 4 mg/kg) • Increased total food intake (1 mg/kg) • Increased perirenal fat pad mass (2 and 4 mg/kg) • No change in fasting glucose levels • Increased fasting insulin, HOMA-IR (1 mg/kg)
Cooper *et al.* (2008) [36]	• Female Wistar rats	• Clozapine I.P.; 1,2,4 mg/kg/day Twice daily 20 days • Clozapine; I.P.; 0.25–0.5 mg/kg	• Weight loss • No change in food intake, muscle mass, levels of glucose, insulin • Increased visceral adiposity
Ersland *et al.* (2015) [57]	Female Sprague Dawley rats	• Olanzapine; intramuscular injection; 100 mg/kg; Once • Risperidone; intramuscular injection; 30 mg/kg; Once • Olanzapine; intramuscular; 10 mg/kg; 3 doses; 35 days • Risperidone; intramuscular; 15 mg/kg; 3 doses; 35 days	• Single treatment: Increased food intake and weight gain • Repeated dosing: Increased food intake and weight gain
Ersland *et al.* (2019) [37]	Female Sprague Dawley rats	Olanzapine; intramuscular; 100 mg/kg; 13 months	Increased body weight gain; Glucose intolerance, insulin intolerance
Fell *et al.* (2004) [38]	Female hooded-Lister rats	Olanzapine; I.P.; 0.5–4 mg/kg 21 days	• Increased body weight and food intake • Increased intra-abdominal fat • No effect on wet/dry uterine weights
Ferno *et al.* (2011) [51]	Female Sprague-Dawley rats	Olanzapine; oral gavage; 3 mg/kg Twice daily	• Induced body weight gain • Increased food intake • Visual decrease in locomotor activity • Weight gain was not different from vehicle in pair-fed animals
Goudie *et al.* (2002) [39]	Female Wistar rats	Olanzapine; B.I.D.; 4 mg/kg 20 days	• Increased body weight • No effect of baseline body weight on weight gain response
Hu *et al.* (2014) [40]	Female Sprague-Dawley rats	Olanzapine; 4 mg/kg 7 days + 8 mg/kg; 7 days	• Increased weight gain, adiposity and food intake compared to vehicle treated rats • Olanzapine decreased BAT weight
Lykkegaard *et al.* (2008) [41]	Female Sprague-Dawley rats	Olanzapine; S.C. mini pumps; 1.75 mg/24 h; 28 days	• Increased food intake, body weight, adiposity • Impaired glucose tolerance (OGTT)
Mann *et al.* (2013) [42]	Famale Sprague-Dawley rats	Olanzapine; osmotic minipump, S.C., i.p.; 7.5 mg/kg/day 14 days	• Increase in body weight • increased food intake • Increased adiposity
Pouzet *et al.* (2003) [43]	Female Mol:Wistar Hannover rats	• Haloperidol; oral gavage; 0.08 and 0.31 mg/kg/day • Olanzapine; oral; 5.0 and 20 mg/kg/day; 21 days	• Haloperidol and olanzapine (5 mg/kg) increased body weight • Olanzapine (20 mg/kg) only significantly increased body weight at 2 weeks; • Olanzapine (5 mg/kg) increased cumulative food intake • All treatments decreased water intake
Skrede *et al.* (2012) [45]	Female Sprague-Dawley rats	• Olanzapine; oral gavage; 6 mg/kg • Aripiprazole; oral gavage; 6 mg/kg 13 days	• Olanzapine increased food intake and weight gain • No effect on fasting glucose or insulin • Olanzapine increased adipose tissue mass
Skrede *et al.* (2014) [44]	Female Sprague-Dawley rats	• Olanzapine; depot injection; 112 mg/kg; Once, Day 0 • Second injection 100 mg/kg Once, day 17 25 day total exposure	• Increased food intake, increased body weight • Decreased hypothalamic AMPKa1 at day 8
Skrede *et al.* (2017) [46]	• Female Sprague Dawley rats • Female OVX Sprague Dawley rats	Olanzapine; intramuscular; 100 mg/kg; Once; 8 days	• Increased food intake and body weight in WT rats • In OVX olanzapine did not potentiate increases in food intake, decreased body weight gain
B. Male Models			
STUDY	MODEL	AP DOSE	EFFECT OF WEIGHT GAIN, FOOD INTAKE, ADIPOSITY METABOLISM
Ader *et al.* (2005) [47]	Male Dogs	• Olanzapine, oral; 15 mg/day • Risperidone; oral; 5 mg/day 21 days	• Olanzapine decreased fasting glucose and insulin compared to baseline • No significant body weight gain or food intake compared to placebo (olanzapine and risperidone) • Olanzapine significantly increased adiposity and decreased insulin sensitivity/decreased hepatic insulin sensitivity • Beta-cell insulin secretion/function failed during graded hyperglycemic test (olanzapine)
Albaugh *et al.* (2006) [33]	Male Wistar and Sprague-Dawley rats	Olanzapine; oral; 4–8 mg/kg 14 days	• No change in body weight or food intake
Albaugh *et al.* (2011) [48]	Male, SpragueDawley rats	Olanzapine; oral in food; ramped dosing 4 mg/kg-20 mg/kg 20 days	• Increased fat mass • Did no effect on body weight or food intake • Increased fasting glucose • Decreased glucose and insulin tolerance
Baptista *et al.* (1993) [49]	Male rats	• Olanzapine; mini-pumps; 5 mg/kg/day • Clozapine; mini pumps; 10 mg/kg/day 11 days	No effect on food intake or body weight in male rats
Cooper *et al.* (2007) [50]	Male Han Wistar rats	Olanzapine; I.P.; 1,2,4 mg/kg/day Twice daily 20 days	• Decreased body weight and no effect on food intake • Enhanced visceral adiposity and reduced lean muscle mass • No increase in plasma levels of insulin or glucose, decreased testosterone levels
Ferno *et al.* (2015) [51]	Male Sprague Dawley rats	• Olanzapine; depot injection; 100 mg/kg Once • Second injection 100 mg/kg Once, day 9 17 day total exposure	• Transient hyperphagia (~7 days) • Weight loss • Increased mesenteric WAT and liver weight • Decreased body weight compared to vehicle treated HFD-fed rats • Worsened adiposity compared to HFD fed rats
Houseknecht *et al.* (2007) [60]	Male Wistar Han rats	Clozapine; S.C.; 10 mg/kg; 5 days	• Increased insulin resistance (HIEC with 5th dose) • Changes in body weight not reported
Minet-Ringuet *et al.* (2006) [52]	Male Sprague-Dawley rats	• Olanzapine; oral with food; 0.01, 0.1, 0.5, 2 mg/kg; 6 weeks • Haloperidol; oral with food; 1 mg/kg; 3 weeks • Olanzapine; oral with food, 1 mg/kg; 3 weeks • Ziprasidone; oral with food; 10 mg/kg; 3 weeks	• Olanzapine (6 weeks): 0.5 mg/kg, 2 mg/kg increased body weight • Matched by increase in subcutaneous adipose tissue • Olanzapine (3 weeks) but not haloperidol or ziprasidone increased body weight and energy intake
Minet-Ringuet *et al.* (2006) [53]	Male Sprague-Dawley rats	• Haloperidol; oral in food; 1 mg/kg • Olanzapine; oral in food; 1 mg/kg. • Ziprasidone; oral in food; 10 mg/kg 6 Weeks	• No effect of treatment on body weight • Haloperidol and olanzapine increased adiposity (s.c. and retroperitoneal WAT) • Haloperidol increased retroperitoneal BAT • Olanzapine increased intrascapular BAT • No effect on cumulative caloric intake or food selection
Pouzet *et al.* (2003) [43]	Male Mol:Wistar Hannover rats	• Haloperidol; oral gavage; 0.08 and 0.31 mg/kg/day • Olanzapine; oral; 5.0 and 20 mg/kg/day 21 days	• Olanzapine (20 mg/kg) decreased body weight • All other groups had no effect on body weight • No effect of treatments on food intake
Smith *et al.* (2008) [54]	Male Sprague dawley rats	• Haloperidol; 0.25 mg/kg • Quetiapine;10 mg/kg • Clozapine;10 mg/kg 28 days	• Haloperidol and clozapine decreased body weight compared to vehicle treatment, no change in fat percentage • Quetiapine showed no difference in body weight, but 40% increase in fat pad mass
Smith *et al.* (2009) [55]	Male Sprague dawley rats (chow + HFD)	Clozapine (10 mg/kg), quetiapine (10 mg/kg) S.C.; 42 days	• Quetiapine alone increased adiposity, no additive effect with HFD • Clozapine alone decreased adiposity; no additive effect of HFD • both quetiapine and clozapine increased blood glucose levels 1-h post treatment at 42 days of treatment • worsened glucose tolerance via GTT, additive to HFD

AP: antipsychotic, OGTT: oral glucose tolerance test, HIEC: Hyperinsulinemic-euglycemic clamp, WAT: white adipose tissue, BAT: brown adipose tissue, HFD: high fat diet, GTT: glucose tolerance test.

**Table 3.** Acute clinical studies examining changes in glucose metabolism in healthy participants in response to antipsychotic treatment.

AUTHORS	PARTICIPANTS	AP TREATMENT	EFFECT ON GLUCOSE METABOLISM
Albaugh *et al.* (2011) [12]	Male (8) + Female (7) Mean age: 26.6	Olanzapine; 10 mg/kg; 3 days	• Worsened glucose tolerance (OGTT) • No change in serum insulin in response to glucose bolus
Hahn *et al.* (2013) [11]	Male (12) + Female (3) Mean age: 30.13	Olanzapine; oral; 10 mg Once	• Increased fasting glucose • Decreased glucose effectiveness
Kopf *et al.* (2012) [72]	Male (10) Age: 24–30 (no mean)	Olanzapine; Oral; 10 mg Once	• No effect on insulin sensitivity (euglycemic-hyperinsulinemic clamp) • Insulin secretion remained intact (hyperglycemic clamp, insulin levels, c-peptide levels)
Sowell *et al.* (2002) [70]	• Olanzapine • Male (13) + Female (4); Mean age: 33.1 • Risperidone • Male (13), Female (0); Mean age: 28.0	• Olanzapine; oral; 10 mg/day • Risperidone; oral; 4 mg/day 15–17 days	• Increase in body weight • Increase in fasting insulin/c-peptide levels, no change in fasting blood glucose • Insulin response/secretion remained intact (hyperglycemic clamp) • Slight decrease in insulin sensitivity (insulin sensitivity index via hyperglycemic clamp)
Sowell *et al.* (2003) [71]	Males + Females (Olanzapine 77.3% males; Risperidone 57.1% males, placebo 68.4% males) Mean age: 33.5	• Olanzapine; oral; 10 mg/day • Risperidone; oral; 4 mg/day 21–23 days	• Increased weight gain with AP treatment • No change in insulin sensitivity (HIEC) • No difference in glucose tolerance or insulin secretion in response to mixed-meal tolerance test
Sacher *et al.* (2008) [73]	Males (35) Mean age: 24.0	• Olanzapine; oral; 10 mg/day • Ziprasidone; oral; 80 mg/day 10 days	• Olanzapine increased BMI • Olanzapine decreased whole body insulin sensitivity (HIEC-decreased glucose uptake) • Olanzapine slightly increased serum insulin levels
Teff *et al.* (2013) [69]	Males (21) + Females (9) Mean age: 26.1	• Olanzapine; oral; 5 mg, 2 days, 10 mg; 7 days • Aripriprazole; oral; 5 mg, 2 days, 10 mg; 7 days	• Olanzapine increased fasting insulin compared to placebo, no change in fasting blood glucose • Olanzapine increased post-prandial insulin, GLP-1, glucagon • Olanzapine increased insulin resistance (HIEC) • No change in body weight, physical activity level, total hunger rating
Vidarsdottir *et al.* (2009) [74]	Males (12) Mean age: 25.1	• Olanzapine; oral standard tablets; 10 mg/day • Olanzapine; oral disintegrating tablets; 10 mg/day 8 days	• No change in adiposity • Olanzapine (both formulations) induced insulin resistance (HOMA-IR)
Vidarsdottir *et al.* (2010) [75]	Males (14) Mean age: 25.7	• Olanzapine; oral; 10 mg/day • Haloperidol; oral; 3 mg/day 8 days	• Olanzapine had no effect on fasting glucose, insulin • Olanzapine decreased insulin sensitivity (HIEC) • Decreased insulin-stimulated glucose disposal • Olanzapine increased blood glucagon, prolactin

AP: antipsychotic, OGTT: oral glucose tolerance test, HIEC: Hyperinsulinemic-euclycemic clamp.

**Table 4.** Acute preclinical studies examining direct effects of antipsychotics on glucose metabolism.

A. Female Models			
STUDY	MODEL	AP DOSE	EFFECT ON GLUCOSE METABOLISM
Albaugh *et al.* (2006) [33]	Female Sprague-Dawley rats	Olanzapine; oral gavage 4 mg/kg 4 doses over 29 h	• Basal fasting glucose was significantly decreased by olanzapine • Olanzapine improved glucose tolerance (via OGTT administered 1-h after last dose) • Matched by significant increase in serum insulin in response to glucose bolus (attenuated compared to vehicle treated rats)
Boyda *et al.* (2012) [76]	Female Sprague-Dawley rats	Olanzapine; I.P. 7.5, 15 mg/kg, Once	• Increased fasting glucose levels, increased serumin insulin levels • Significantly impaired glucose tolerance (ipGTT)
Boyda *et al.* (2014) [77]	Female Sprague-Dawley rats	Olanzapine; I.P.; 7.5, 15 mg/kg; Once	• No effect on fasting blood glucose levels • Significantly increased fasting serum insulin • Olanzapine decreased glucose tolerance (7.5, 15 mg/kg; ipGTT)
Houseknecht *et al.* (2007) [60]	Female CD/Sprague-Dawley Rats	• Olanzapine; S.C. • 10 mg/kg; Once (60 min before dissection/*ex vivo* incubation) • Clozapine; S.C.; 10 mg/kg; Once (60 min before dissection/*ex vivo* incubation)	• No effect on in vitro basal or insulin-stimulated glucose uptake in isolated epitrochlearis muscle
Jassim *et al.* (2012) [78]	Female Sprague-Dawley rats	• Olanzapine; I.P.; 5 mg/kg • Clozapine; I.P.; 25 mg/kg	• Increased serum glucose approximately 1 h posttreatment (olanzapine increased approximately 1 mmol/L (resolved by next time point-3 h)) • Olanzapine: No significant effect on serum insulin, or serum glucagon, slightly decreased gluconeogenic precursors • Clozapine: increased glucagon 30 min posttreatment, increased gluconeogenic precursors
Martins *et al.* (2010) [79]	Female C57/BL6 Mice	Olanzapine; I.P.; 4.5 mg/kg Once	• Olanzapine increased blood glucose • Serum insulin levels not reported
Wu *et al.* (2014) [80]	Female Sprague-Dawley Rats	Olanzapine; S.C. 10 mg/kg (for ipGTT) 1.5 mg/kg, 15 mg/kg (for HIEC) Once	• No effect on fasting (overnight) blood glucose • Large increase in fasting insulin levels • Decreased glucose tolerance (ipGTT) • High dose (15 mg.kg) but not low dose (1.5 mg/kg) increased insulin resistance (HIEC)
B. Male Models			
STUDY	MODEL	AP DOSE	EFFECT ON GLUCOSE METABOLISM
Albaugh *et al.* (2011) [48]	Male Sprague-Dawley rats	Olanzapine; Oral gavage 10 mg/kg 2 Doses (PM + AM 2 h preceding experiments)	• Increased blood glucose 2 h post-treatment in fed and fasted (5 h, 15 h) rats • Increased insulin in fasted state, no affect in fed state • Impaired insulin sensitivity (HIEC) but insulin signaling remained intact in response to hyperinsulinemia
Albaugh *et al.* (2012) [81]	Male Sprague-Dawley rats	Olanzapine; Oral gavage 4 mg/kg 2 doses (PM and AM 1 h preceding experiment)	• Increased fasting glucose levels • Decreased glucose tolerance (OGTT) and insulin tolerance (ITT) • Plasma insulin did not change during OGTT or in response to olanzapine
Bush *et al.* (2018) [82]	Male C57BL6J mice	Olanzapine; I.P. 5 mg/kg; Once	• Increased blood glucose immediately posttreatment Prevented via activation of liver AMPK (via AICAR)
Castellani *et al.* (2017) [83]	Male C57BL6J mice + male Grgrc−/−mice	Olanzapine; I.P. 5 mg/kg; Once	• Increased blood glucose immediately posttreatment • No change in serum insulin, increased serum glucagon • Glucagon receptor knockout mice protected from olanzapine-induced increase in blood glucose, but not olanzapine-induced reductions insulin tolerance
Castellani *et al.* (2018) [84]	Male C57BL6J mice	Olanzapine; I.P.; 5 mg/kg; Once	• Increased blood glucose immediately post-treatment, no change in serum insulin
Chintoh *et al.* (2008) [85]	Male Sprague-Dawley rats	Olanzapine; S.C. 3 mg/kg; Once	• Increased blood glucose • Decreased insulin release in response to glucose bolus (lower insulin and c-peptide levels during hyperglycemic clamp) • Increased insulin resistance (HIEC-increased glucose production/decreased glucose uptake)
Chintoh *et al*., (2009) [86]	Male Sprague-Dawley rats	• Clozapine; S.C.; 10 mg/kg • Olanzapine; S.C.; 3.0 mg/kg • Risperidone; S.C.; 1 mg/kg • Ziprasidone; S.C.; 3 mg/kg • Haloperidol; S.C.; 0.25 mg/kg Once	• Olanzapine and clozapine decreased insulin sensitivity (HIEC), increased hepatic glucose production • Olanzapine and clozapine decreased insulin secretion in response to glucose bolus (hyperglycemic clamp)
Girault *et al.* (2012) [87]	Male Wistar Han rats	• Olanzapine; intragastirc; 3 mg/kg/h; Once • Olanzapine; ICV; 30 mg/kg/h, Once	• Increased blood glucose levels, no change in plasma insulin, corticosterone • ICV olanzapine had no effect on blood glucose levels or endogenous glucose production, insulin, corticosterone levels
Hahn *et al.* (2014) [88]	Male Sprague-Dawley rats	Olanzapine; ICV; 75 ug; Once	• Decreased insulin secretion (decreased insulin and c-peptide levels during hyperglycemic clamp) • Increased blood glucose levels (lower glucose infusion rate to reach hyperglycemia in response to olanzapine treatment) • No effect on insulin resistance (HIEC)
Houseknecht *et al.* (2007) [60]	Male Wistar Han rats	• Clozapine; S.C.; 1, 3.2, 10 mg/kg; Once • Olanzapine; S.C.; 1, 3.2, 10 mg/kg; Once • Risperidone; S.C.; 2 mg/kg; Once • Ziprasidone; S.C.; 3.2, 10, 32 mg/kg; Once	Clozapine (3.2, 10 mg/kg): Increased insulin resistance (HIEC) • Olanzapine (3.2, 10 mg/kg): Increased insulin resistance (HIEC) • Risperidone: No effect on insulin sensitivity (HIEC) • Ziprasidone: No effect on insulin sensitivity (HIEC)
Ikegami *et al.* (2013) [89]	Male ICR Mice	Olanzapine; ICV; 5, 10, 15 nmol; Once	• Dose dependent increase in blood glucose at doses of 10 and15 nmol • No change in blood insulin
Ikegami *et al.* (2013) [90]	Male ICR mice	• Olanzapine; I.P; 0.3,1,3,5 mg/kg, Once • Olanzapine; ICV; 10, 15 nmol; Once	• I.P. dose (3,5 mg/kg): Decreased glucose tolerance (ipGTT) • ICV dose: Decreased glucose tolerance (ipGTT) Changes involve activation of hypothalamic AMPK • No effect of olanzapine on insulin or glucagon levels (20 min post-treatment)
Ikegami *et al.* (2013) [91]	Male ICR mice,6 weeks old	• Olanzapine; I.P; 0.3,1,3,5 mg/kg, Once • Olanzapine; ICV; 10, 15 nmol; Once	• I.P. treatment increased blood glucose in fed and overnight fasted mice • ICV treatment increased blood glucose in fed mice only • Involves AMPK activation at hypothalamus and beta-adrenergic activation
Klingerman *et al.* (2015) [92]	Male Sprague-Dawley rats	• Olanzapine; ICV (3rd ventricle); 0.1, 0.2, 0.3, 0.44 mg/kg • Olanzapine; ICV (lateral ventricle); 0.44, 1.8 mg/kg • Olanzapine; Oral gavage; 0.44, 1, 3,10 mg/kg)	• ICV: Increased blood glucose 30 min posttreatment to 3rd ventricle (0.3 mg/kg, 0.44 mg/kg) • ICV: Increased blood glucose 30 min posttreatment to lateral ventricle (1.8 mg/kg) • Increased blood glucose in fasted (4-h) rats 30 min post-treatment (3 mg/kg, 10 mg/kg) and 1 h post-treatment (10 mg/kg)
Kowalchuk *et al.* (2017) [93]	Male Sprague-Dawley rats	Olanzapine; S.C.; 2 mg/kg Once	• No effect of olanzapine during euglycemic-pancreatic clamp (somatostatin to block endogenous insulin secretion, exogenous insulin replaced at basal levels)
Martins *et al.* (2010) [79]	Male Sprague-Dawley Rats	• Olanzapine; primed continuous IV infusion;1 mg/kg/h Once • Olanzapine; ICV; a bolus of 110 g and 73.4 g/h Once	• Increased blood glucose levels, no change in plasma insulin • Increased rate of glucose production, decreased rate of uptake (HIEC) • Increased endogenous glucose production at liver and increased p-AMPK at hypothalamus
Nagata *et al.* (2016) [94]	Male Wistar Rats	Olanzapine; I.V.; 2.5, 5, 10 mg/kg Once	• High dose of olanzapine (10 mg/kg) increased blood glucose and blood insulin (max ~1 h post-AP) • Increased blood cortisol but no change in serum glucagon • Effect blocked by treatment with adrenergic receptor antagonist propranolol
Smith *et al.* (2008) [54]	Male Sprague-Dawley rats	• Haloperidol; 0.25 mg/kg • Quetiapine;10 mg/kg • Clozapine;10 mg/kg 28 days	• Blood glucose and plasma insulin levels were significantly increased by 1 h after haloperidol, quetiapine or clozapine injections • Decreased glucose tolerance (via decreased glucose clearance in response to quetiapine and clozapine) • Increased hepatic glucose output (PTT)
Townsend *et al.* (2018) [61]	Male C57BL6J mice	Olanzapine; I.P.; 5 mg/kg Once	• Increased blood glucose post-treatment • Potentiated with 4 week high fat feeding • Increased gluconeogenesis, decreased insulin sensitivity and signaling

AP: antipsychotic, iPGTT: intraperitoneal glucose tolerance test, HIEC: hyperinsulinemic-euglycemic clamp, PTT: pyruvate tolerance test.
